# Genomic, immunologic, and prognostic associations of TROP2 (*TACSTD2*) expression in solid tumors

**DOI:** 10.1093/oncolo/oyae168

**Published:** 2024-07-10

**Authors:** Dan Morgenstern-Kaplan, Samuel A Kareff, Asaad Trabolsi, Estelamari Rodriguez, Harris Krause, Jennifer R Ribeiro, Heng Tan, Emmanuel S Antonarakis, Emil Lou, Misako Nagasaka, Sandra Algaze, Heinz-Josef Lenz, Stephen V Liu, Balazs Halmos, Dave S B Hoon, Andreas Seeber, Patrick C Ma, Wafik S El-Deiry, Ari M Vanderwalde, Gilberto Lopes

**Affiliations:** Department of Medicine, Division of Medical Oncology, University of Miami Sylvester Comprehensive Cancer Center/Jackson Memorial Hospital, Miami, FL 33131, United States; Department of Medicine, Division of Medical Oncology, University of Miami Sylvester Comprehensive Cancer Center/Jackson Memorial Hospital, Miami, FL 33131, United States; Department of Medicine, Division of Medical Oncology, University of Miami Sylvester Comprehensive Cancer Center/Jackson Memorial Hospital, Miami, FL 33131, United States; Department of Medicine, Division of Medical Oncology, University of Miami Sylvester Comprehensive Cancer Center/Jackson Memorial Hospital, Miami, FL 33131, United States; Caris Life Sciences, Phoenix, AZ 85040, United States; Caris Life Sciences, Phoenix, AZ 85040, United States; Department of Medicine, Division of Medical Oncology, University of Miami Sylvester Comprehensive Cancer Center/Jackson Memorial Hospital, Miami, FL 33131, United States; University of Minnesota Masonic Cancer Center, Minneapolis, MN 55455, United States; University of Minnesota Masonic Cancer Center, Minneapolis, MN 55455, United States; Division of Hematology/Oncology, University of California Irvine School of Medicine, Orange, CA 92617, United States; Division of Medical Oncology, Keck School of Medicine of University of Southern California, Los Angeles, CA 90033, United States; Division of Medical Oncology, Keck School of Medicine of University of Southern California, Los Angeles, CA 90033, United States; Georgetown Lombardi Comprehensive Cancer Center, Georgetown University, Washington, DC 20007, United States; Montefiore Einstein Comprehensive Cancer Center, Bronx, NY 10461, United States; Saint John’s Cancer Institute, Providence Health System, Santa Monica, CA 90404, United States; Tyrolean Cancer Research Institute, Innsbruck Medical University, Innsbruck 6020, Austria; Division of Hematology/Oncology, Penn State Cancer Institute, Hershey, PA 17033, United States; Legorreta Cancer Center, Warren Alpert Medical School of Brown University, Providence, RI 02912, United States; Caris Life Sciences, Phoenix, AZ 85040, United States; Department of Medicine, Division of Medical Oncology, University of Miami Sylvester Comprehensive Cancer Center/Jackson Memorial Hospital, Miami, FL 33131, United States

**Keywords:** TROP2, precision oncology, targeted therapy, tumor genetics

## Abstract

**Background:**

TROP2 (*TACSTD2*) expression is associated with decreased overall survival (OS) in some solid tumors, and the TROP2-targeting antibody-drug conjugate (ADC) sacituzumab govitecan has been approved in breast and urothelial carcinomas. We aimed to explore the multi-omic landscape associated with *TACSTD2* gene expression in various solid tumors to identify patients most likely to benefit from this approach.

**Methods:**

Breast (*N* = 11 246), colorectal (*N* = 15 425), hepatocellular (*N* = 433), pancreatic (*N* = 5488), and urothelial (*N* = 4125) tumors were stratified into quartiles by *TACSTD2* gene expression, analyzed by next-generation DNA sequencing, whole transcriptome sequencing, and immunohistochemistry at Caris Life Sciences (Phoenix, AZ). Survival data were obtained from insurance claims, and Kaplan-Meier estimates were calculated for molecularly defined cohorts.

**Results:**

Several pathogenic mutations were associated with *TACSTD2*-high tumors, including *TP53* in breast, colorectal (CRC), pancreatic, and hepatocellular cancers; *KRAS* in pancreatic and CRC cancers; *ARID1A* and *FGFR3* in urothelial cancer; and *CTNNB1* in hepatocellular cancer. *TACSTD2-low* breast tumors were enriched for copy number amplifications in *CCND1* and *FGF/R* family member genes. *TACSTD2* high was generally associated with more immune cell infiltration and greater T-cell inflammation scores. Patients with *TACSTD2*-high breast, CRC, and pancreatic cancers demonstrated a significantly shorter OS than *TACSTD2*-low tumors. This was restricted to CRC with microsatellite stable tumors and patients with pancreatic cancer with *KRAS*-mutant tumors. Patients with breast cancer with *TACSTD2*-high tumors also experienced significantly worse OS following immune checkpoint inhibitors.

**Conclusions:**

*TACSTD2* expression is associated with key driver alterations and a more active immune microenvironment, suggesting possible combinatorial strategies with TROP2-targeting ADCs plus immunotherapy in various solid tumors.

## Teaser

The TROP2-targeting antibody-drug conjugate sacituzumab govitecan has been approved in breast and urothelial carcinomas. This study explored the multi-omic landscape associated with TACSTD2 gene expression in various solid tumors to identify patients most likely to benefit from this approach.

**Implications for Practice**

This study reveals that *TACSTD2*-high tumors are largely associated with a worse prognosis, specifically among patients with breast cancer, patients with pancreatic cancer with *KRAS*-mutant tumors, and patients with CRC with microsatellite stable tumors. *TACTSTD2*-high tumors were enriched in specific driver alterations in each tumor type. TACSTD2-high tumors also displayed transcriptomic signatures indicating greater immune cell infiltration and T-cell inflammation scores. Clinically, these results suggested that combinatorial strategies involving immune checkpoint inhibitors and/or other targeted therapies may be considered along with TROP2-targeting antibody-drug conjugates in *TACSTD2*-high solid tumors.

## Introduction

Trophoblastic cell-surface antigen 2 (TROP2) is a transmembrane glycoprotein originally identified as an antigen in trophoblasts of the placenta aiding in implantation.^1^ More recently, it has been implicated in cancer signaling as well, giving rise to its gene name Tumor-associated calcium signal transducer 2 (*TACSTD2*).^1^ TROP2/*TACSTD2* is elevated in tumor tissue relative to normal and therefore has gained interest as a cancer-specific drug target, particularly for late-stage disease.^2^

Overexpression of TROP2/*TACSTD2* has been shown to confer a poor prognosis, notably among patients with gynecologic or gastrointestinal tumors.^3^ Its physiologic functions may be subverted to aid in proliferation and metastasis of tumor cells through various pathways such as insulin-like growth factor-1 (IGF-1) and extracellular regulated kinase-1/2 (ERK1/2).^1^ TROP2/*TACSTD2* has also been shown to have some impact on the tumor immune microenvironment and may modulate immunotherapy response. In cervical cancer, TROP2 levels were associated with increased intratumoral tumor-infiltrating lymphocytes and programmed cell death-ligand 1 (PD-L1) expression.^4^ Conversely, TROP2/*TACSTD2*-high tumors had lower gene expression of immune cell markers in breast and non–small cell lung cancer,^5,6^ and were independently associated with poor response to immune checkpoint inhibitors (ICIs).^6^ These results highlight tumor-type differences in the TROP2-mediated immune landscape.

As of 2023, the results of several TROP2-targeting clinical trials in advanced solid tumors have been published, with most of these investigating the efficacy of sacituzumab govitecan (SG), an antibody-drug conjugate (ADC) that delivers an SN38 toxic payload (a metabolite derived from irinotecan) to tumor cells.^1,7^ In the phase 3 ASCENT trial for patients with metastatic triple negative breast cancer (TNBC), a 35% objective response rate (ORR) was observed compared to 5% for the control arm.^8^ Likewise, the phase 2 TROPHY-U-01 trial for metastatic, pretreated patients with urothelial carcinoma showed a 27% ORR.^9^ The results of these trials led to FDA approvals of SG in these disease settings. Most recently, the phase 3 TROPiCS-02 trial found that SG yielded a 21% ORR versus 14% for systemic chemotherapy in hormone receptor-positive (HR+)/HER2-negative (HER2−) metastatic breast cancer.^10^

Analysis of the TROPiCS-02 trial revealed that therapeutic response to SG was achieved in patients with breast cancer with both low or high TROP2 (assessed by immunohistochemistry [IHC]),^11^ but most of the aforementioned studies failed to stratify patients by TROP2 level. However, one study in CRC found superior benefit from chemotherapy intensification in patients with medium/high TROP2 levels, and preclinical studies show the selective response of TROP2-expressing cells to SG.^12-14^ Furthermore, the COGNITION-GUIDE trial (NCT05332561) is currently enrolling patients with high-risk early breast cancer to test the efficacy of adjuvant targeted therapies including SG for TROP2-positive tumors. Therefore, TROP2 expression has the potential to become a therapeutic biomarker.^15^

In the present study, we explored the genomic, transcriptomic, and immunologic landscape associated with *TACSTD2* gene expression in several solid tumor types using a real-world cancer cohort of 36 717 patients. Pan-cancer characterization of the molecular landscape associated with *TACSTD2* may help expand the application of TROP2-targeting agents into additional tumor types and identify possible therapeutic cotargets.

## Materials and methods

### Cohort information

A total of 198 394 tumor specimens underwent comprehensive molecular profiling at Caris Life Sciences (Phoenix, AZ, USA) including breast cancer (*N* = 11 246), colorectal cancer (CRC; *N* = 15 425), liver cancer (*N* = 433), pancreatic ductal adenocarcinoma (*N* = 5488), and urothelial cancer (*N* = 4125) were included in the study.

### Defining tumor site

A primary (local) tumor label was assigned when the primary and biopsy specimen sites were from the same organ. A metastatic tumor was defined as any nonprimary tumor. Breast tumors were divided based on hormone receptor subtype. For urothelial cancer, upper and lower urothelial tract were defined by the annotated primary site and biopsy specimen site. Upper corresponds to tumors arising in the kidney and ureter while lower refers to tumors arising in the urinary bladder and/or urethra.

### Next-generation DNA sequencing

Next-generation DNA sequencing (NGS) was performed on 36 717 tumors. Prior to molecular testing, tumor enrichment was achieved by harvesting targeted tissue using manual microdissection techniques, with a minimum of 20% tumor content. Genomic DNA was isolated from formalin-fixed paraffin-embedded (FFPE) tumor samples. Sequencing was performed using a custom-designed SureSelect XT assay (592-gene targets; Agilent Technologies, Santa Clara, CA, USA) on the NextSeq platform (Illumina, Inc., San Diego, CA, USA) or by whole-exome sequencing (WES) on the Illumina NovaSeq 6000 platform (Illumina, Inc.). For WES, a hybrid pull-down panel of baits designed to enrich for 700 clinically relevant genes at high coverage and high read-depth was used, along with another panel designed to enrich for additional >20 000 genes at lower depth. The performance of the 592 and WES assays was validated for copy number amplification (CNA). Matched normal tissue was not sequenced.

### Identification of genetic variants

Genetic variants identified were interpreted by board-certified molecular geneticists and categorized as “pathogenic,” “likely pathogenic,” “variant of unknown significance,” “likely benign,” or “benign,” according to the American College of Medical Genetics and Genomics (ACMG) standards. When assessing mutation frequencies of individual genes, “pathogenic,” and “likely pathogenic” were counted as mutations, while “benign,” “likely benign” variants, and “variants of unknown significance” (VUS) were excluded.

### Whole transcriptome sequencing

FFPE specimens underwent pathology review to diagnose percent tumor content and tumor size; a minimum of 10% of tumor content in the area for microdissection was required to enable enrichment and extraction of tumor-specific RNA. Qiagen RNA FFPE kit (Qiagen, Germantown, MD, USA) was used, and the RNA quality and quantity was determined using the Agilent TapeStation (Agilent Technologies). Biotinylated RNA baits were hybridized to the synthesized and purified cDNA targets and the bait-target complexes were amplified in a post-capture PCR reaction. The resultant libraries were quantified and normalized, and the pooled libraries were denatured, diluted, and sequenced. For transcript counting, transcripts per million molecules (TPM) were generated using the Salmon expression pipeline. *TACSTD2*-high and *TACSTD2*-low expression were defined as ≥top and <bottom quartile of *TACSTD2* TPM, respectively (breast cancer Q1: 0.0-32.9 TPM, Q4: 122.8-1721.6 TPM; CRC Q1: 0-2.9, Q4: 24.4-808.4 TPM; liver cancer Q1: 0-0.3, Q4: 2.3-197.2 TPM; pancreatic ductal adenocarcinoma Q1: 0-44.8, Q4: 154.1-1845.6 TPM; and urothelial cancer Q1: 0-96.6, Q4: 333.6-3640.2 TPM).

### Immune signatures

Immune cell fraction was inferred via deconvolution of whole transcriptome sequencing (WTS) data by quanTIseq.^16^ QuanTIseq is an immune deconvolution algorithm that uses RNA transcripts that are known to be expressed in specific immune cell types to deconvolute bulk RNA sequencing data and predict the different immune cell fractions that were present in the bulk RNA lysate. WTS data were also used to calculate a T-cell-inflamed score as previously described.^17^

### Microsatellite instability/mismatch repair deficiency (MSI-H/dMMR) status

A combination of multiple test platforms was used to determine MSI-H/dMMR status of the tumors profiled, including fragment analysis (FA, Promega, Madison, WI, USA); IHC for MLH1 (M1 antibody), MSH2 (G2191129 antibody), MSH6 (44 antibody), and PMS2 (EPR3947 antibody; Ventana Medical Systems, Inc., Tucson, AZ, USA); and NGS (for tumors tested with NextSeq platform, 7000 target microsatellite loci were examined and compared to the reference genome hg19 from the University of California, Santa Cruz). The 3 platforms generated highly concordant results as previously reported and in the rare cases of discordant results, the MSI-H or dMMR status of the tumor was determined in the order of IHC, FA, and NGS.

### Tumor mutational burden

Tumor mutational burden (TMB) was measured by counting all nonsynonymous missense, nonsense, inframe insertion/deletion and frameshift mutations found per tumor that had not been previously described as germline alterations in dbSNP151, Genome Aggregation Database (gnomAD) or benign variants identified by Caris geneticists. A cutoff point of ≥10 mutations per MB was used based on the KEYNOTE-158 pembrolizumab trial, which showed that patients with a TMB of ≥10 mt/MB (TMB-H) across several tumor types had higher response rates than patients with a TMB of <10 mt/MB.^18,19^

### Immunohistochemistry

IHC was performed on FFPE sections on glass slides. Slides were stained using automated staining techniques, per the manufacturer’s instructions, and were optimized and validated per CLIA/CAP and ISO requirements. Staining was scored for intensity (0 = no staining; 1+ = weak staining; 2+ = moderate staining; 3+ = strong staining) and staining percentage (0%-100%). PD-L1 (SP142) positive (+) staining was defined as ≥2+ and ≥5%. ER+ or PR+ was defined as ≥1+ and ≥1%. HER2/Neu+ was defined as ≥3+ and >10%.

### Clinical outcomes

Real-world overall survival (OS) was obtained from insurance claims and calculated from either tissue collection, start of ICI (Atezolizumab, Avelumab, Nivolumab or Pembrolizumab), or start of SG to last contact. Kaplan-Meier estimates were calculated for molecularly defined patients.

### Statistical methods

Descriptive analyses were conducted using Mann-Whitney *U* (scipy V.1.9.3) and χ^2^/Fisher-Exact tests (R v.3.6.1) for continuous and categorical variables, respectively. *P* values were adjusted for multiple comparisons with *P* < .05 considered significant.

### Ethics statement

This study was conducted in accordance with the guidelines of the Declaration of Helsinki, Belmont report, and US Common rule. In keeping with 45 CFR 46.101(b)(4), this study was performed using retrospective, deidentified clinical data and is considered institutional review board exempt. No patient consent was necessary from the subjects.

## Results

### Pan-cancer analysis of TACSTD2 expression

**Figure 1.**
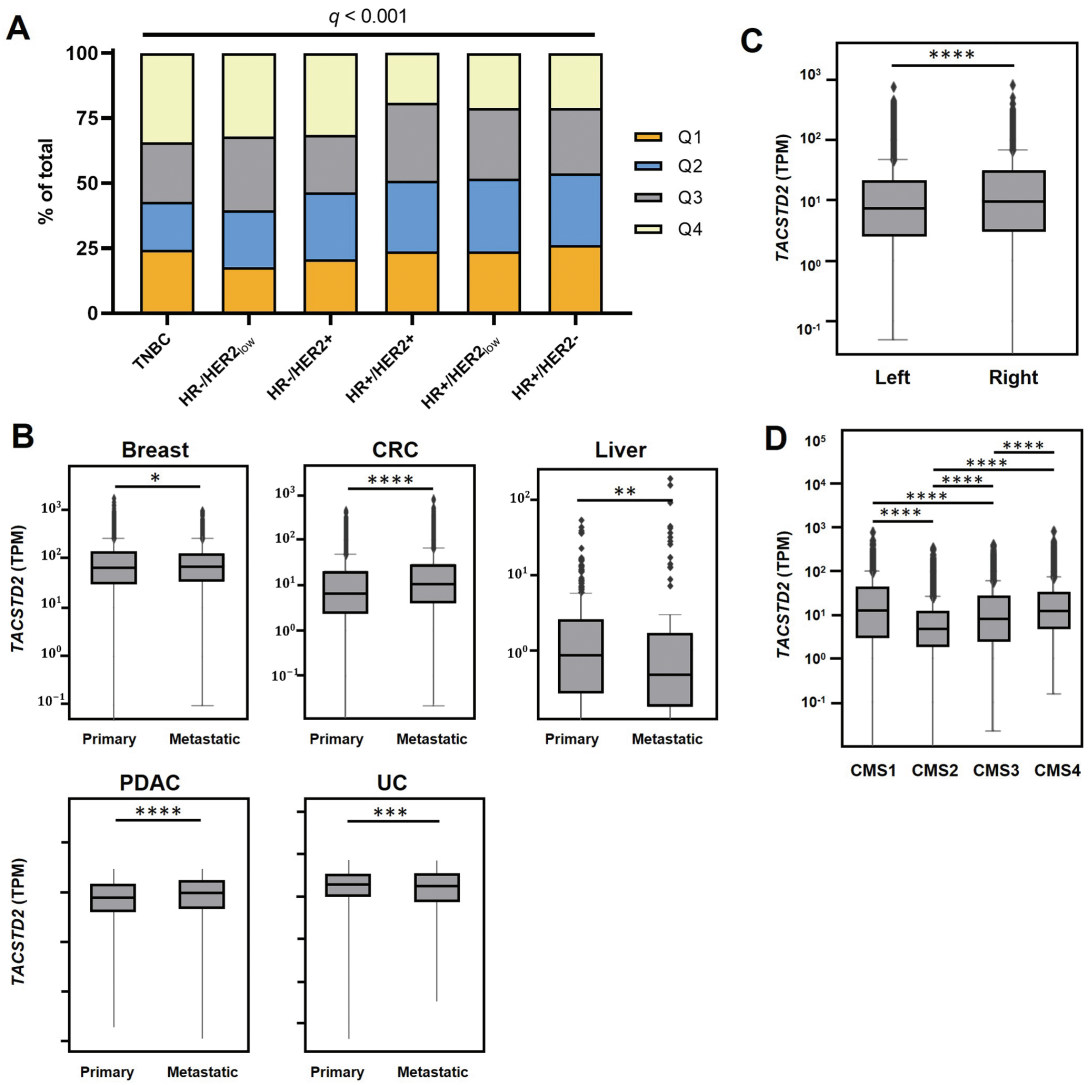
Pan-cancer expression of *TACSTD2*. (A) *TACSTD2* expression in transcripts per million (TPM) between primary and metastatic sites in breast cancer, colorectal, hepatocellular cancer, pancreatic ductal adenocarcinoma, and urothelial carcinoma. (B) *TACSTD2* expression in left- and right-sided colorectal tumors and (C) segmented based on CRC consensus molecular subtype (CMS) (D; *P* < .05).

Demographic and tumor characteristics of the patients segregated by *TACSTD2* expression in quartiles (Q1-Q4) are described in Table 1. The 5 tumor types selected for analysis were based on their representation of a broad expression of *TACSTD2* (Supplementary Figure S1). There was an approximately equal distribution of sex and median age between each quartile (Table 1). There was a greater percentage of *TACSTD2*-high tumors (Q4) among TNBC (34%), HR−/HER2_low_ (32%), and HR−/HER2+ (31%), compared to HR+/HER2+ (19%), HR+/HER2low (21%), and HR+/HER2− (21%) tumors (*P* < .001; Figure 1A). Expression of *TACSTD2* was significantly higher in metastatic sites, compared to the primary tumor in breast, colorectal, pancreatic, and urothelial cancers. Conversely, *TACSTD2* expression was higher in primary liver cancer compared to metastatic (*P* < .005; Figure 1B). In CRC, right-sided tumors had significantly higher expression of *TACSTD2* than left-sided tumors (Figure 1C). When segmented by consensus molecular subtypes (CMS), CMS1 tumors had the highest expression of *TACSTD2*, followed by CMS4, CMS3, and CMS2 (Figure 1D).

**Table 1.** Clinical and demographic information of pan-cancer cohort.

	*TACSTD2* Q1 (lowest)	*TACSTD2* Q2	*TACSTD2* Q3	*TACSTD2* Q4 (highest)	*q* value
Breast cancer					
Count (N)	2812	2811	2811	2812	
Median age [range] (N)	61 [22->89] (2812)	61 [23->89] (2811)	61 [19->89] (2811)	61 [22->89] (2812)	.44
Female	98.5% (2769/2812)	98.6% (2772/2811)	98.7% (2774/2811)	99.0% (2785/2812)	.34
Primary	34.63% (956/2761)	38.94% (1079/2771)	38.48% (1066/2770)	37.77% (1048/2775)	.004
TNBC	25.77% (626/2492)	19.15% (471/2459)	23.63% (590/2497)	35.05% (876/2499)	<.001
HR−/HER2low	3.5% (85/2492)	4.23% (104/2459)	5.41% (135/2497)	6.12% (153/2499)	
HR−/HER2+	2.88% (70/2492)	3.54% (87/2459)	3.00% (75/2497)	4.24% (106/2499)	
HR+/HER2+	4.53% (110/2492)	5.08% (125/2459)	5.53% (138/2497)	3.56% (89/2499)	
HR+/HER2low	19.14% (465/2492)	22.16% (545/2459)	21.19% (529/2497)	16.61% (415/2499)	
HR+/HER2−	44.17% (1073/2492)	45.83% (1127/2459)	41.25% (1030/2497)	34.41% (860/2499)	
Colorectal carcinoma					
Count (N)	3857	3856	3856	3856	
Median age [range] (N)	63 [18->89] (3857)	62 [18->89] (3856)	62 [14->89] (3856)	62 [17->89] (3856)	.01
Female	46.1% (1778/3857)	43.9% (1691/3856)	46.2% (1783/3856)	46.0% (1775/3856)	.11
Primary	68% (2460/3619)	58% (2107/3625)	49% (1801/3655)	51% (1837/3581)	<.001
Liver cancer					
Count (N)	109	108	108	108	
Median age [range] (N)	68 [17-89] (109)	66 [21->89] (108)	66.5 [13->89] (108)	65 [18->89] (108)	.68
Female	22.9% (25/109)	20.4% (22/108)	21.3% (23/108)	26.9% (29/108)	.68
Primary	68% (73/108)	68% (73/108)	78% (83/107)	84% (91/108)	.01
Pancreatic ductal adenocarcinoma					
Count (N)	1372	1372	1372	1372	
Median age [range] (N)	68 [13->89] (1372)	67 [23->89] (1372)	68 [26->89] (1372)	68 [29->89] (1372)	.52
Female	48.8% (669/1372)	47.4% (651/1372)	48.5% (666/1372)	43.9% (602/1372)	.09
Primary	45% (602/1330)	47% (618/1329)	42% (558/1329)	36% (479/1323)	<.001
Urothelial carcinoma					
Count (N)	1032	1031	1031	1031	
Median age [range] (N)	72 [26->89] (1032)	72 [27->89] (1031)	72 [18->89] (1031)	73 [24->89] (1031)	.51
Female	30.7% (317/1032)	29.5% (304/1031)	27.2% (280/1031)	24.4% (252/1031)	.21
Primary	62.43% (643/1030)	65.66% (675/1028)	72.21% (743/1029)	67.48% (695/1030)	<.001

### Landscape of TACSTD2-associated molecular alterations in solid tumors

**Figure 2.**
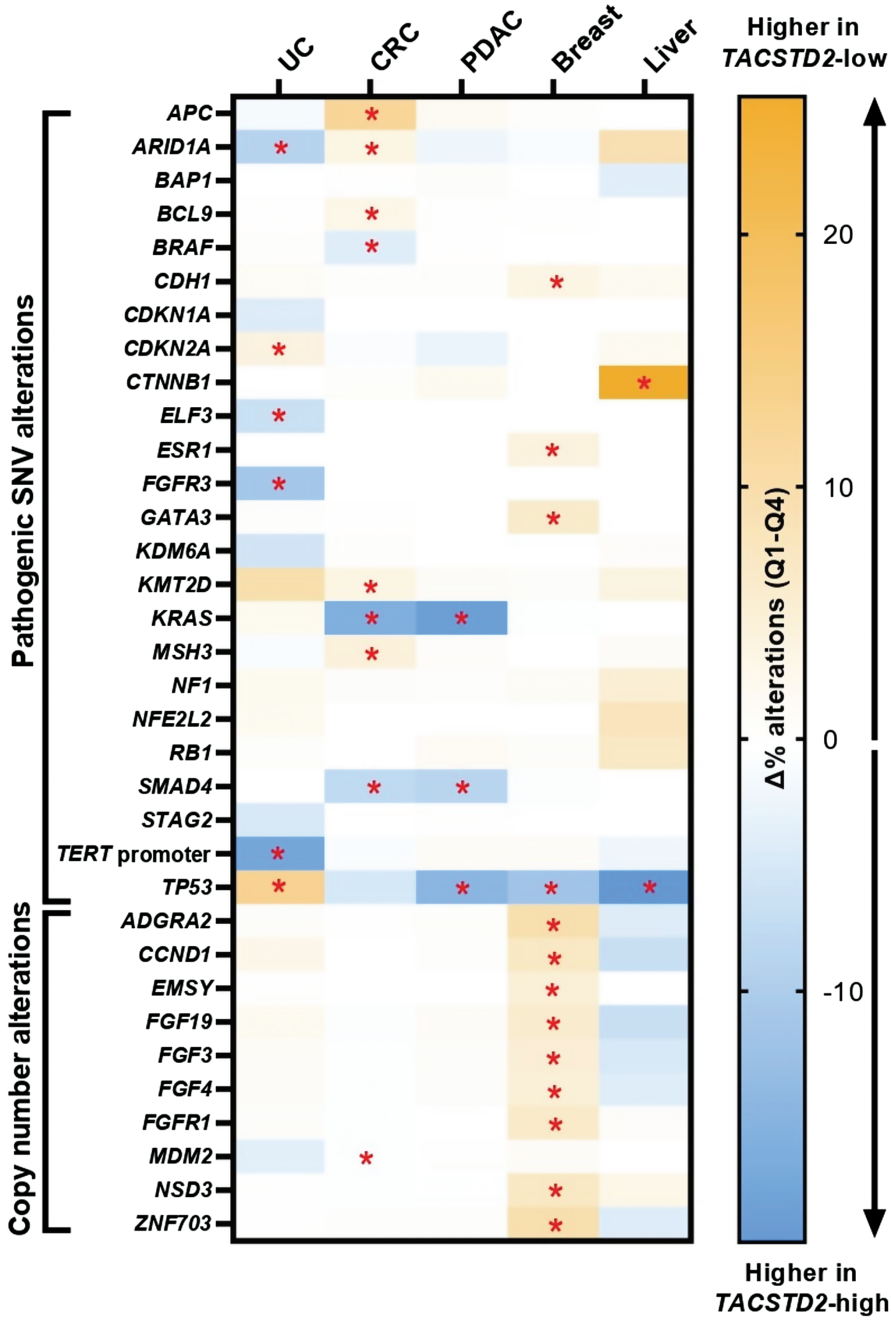
Landscape of *TACSTD2*-associated genomic alterations. The heat map shows the difference in the prevalence of mutations and copy number alterations between *TACSTD2*-high and *TACSTD2*-low tumors. Orange indicates the alteration is associated with *TACSTD2*-low; blue indicates the alteration is associated with *TACSTD2*-high. An alteration is included in the heatmap if it has an absolute difference in prevalence of >3% in one of the investigated cancer types. Red asterisks indicate statistical significance (*q *< .05). Abbreviations: CRC: colorectal cancer; PDAC: pancreatic ductal adenocarcinoma; UC: urothelial carcinoma.

Genomic sequencing revealed that several gene alterations including driver mutations were significantly associated (*P* < .05) with *TACSTD2* expression. Compared to *TACSTD2*-low, there was a greater prevalence of *TP53* mutations in *TACSTD2-*high breast (59% vs 48%), colorectal (77% vs 71%), pancreatic (83% vs 69%), and liver cancer (43% vs 23%), but a lower prevalence in urothelial cancer (38% vs 54%). *TACSTD2*-high tumors also had a significantly higher rate of *KRAS* mutations in pancreatic (96% vs 78%) and CRC (55% vs 38%; Figure 2). Among all pathogenic *KRAS* mutations, the prevalence of G12C was similar between *TACSTD2*-high versus *TACSTD2*-low groups in both CRC (7.3% vs 6.4%) and pancreatic cancer (1.5% vs 1.5%; *P* > .05; data not shown).

Mutations in the genes *ARID1A* and *FGFR3* were also associated with *TACSTD2* expression. In urothelial cancer, *TACSTD2*-high tumors had a higher rate of mutation in *ARID1A* (29% vs 20%) and *FGFR3* (18.7% vs 7.6%), while *ARID1A* mutations were less frequent in *TACSTD2*-high CRC (6.7% vs 10.2%; Figure 2). Strikingly, in hepatocellular cancer, *TACSTD2*-high tumors had a much lower prevalence of *CTNNB1* mutations (25.0% vs 55.5%). Upon examination of copy number alterations (CNAs), only breast cancer demonstrated a significant pattern of differential prevalence based on *TACSTD2* expression. CNAs in several genes including Cyclin D1 (*CCND1*) and fibroblast growth factor/receptor (*FGF/FGFR)* family members were more frequent in *TACSTD2*-low tumors.

### TACSTD2-associated tumor immune landscape

**Figure 3.**
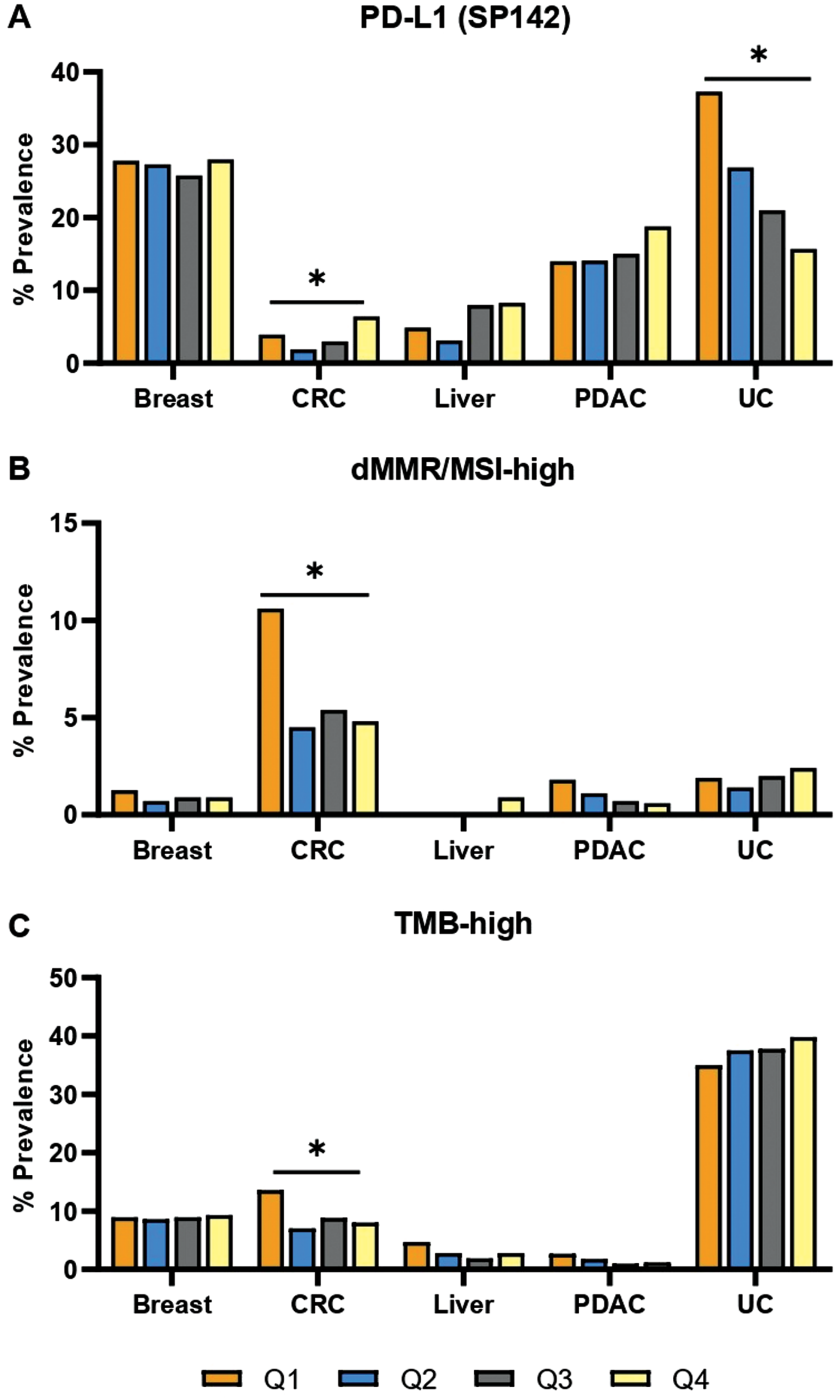
Pan-cancer prevalence of immune-oncology biomarkers stratified by *TACSTD2* expression. Prevalence of PD-L1 positivity by immunohistochemistry (A), mismatch repair deficiency/microsatellite instability-high (dMMR/MSI-high) (B), and tumor mutational burden-high (TMB-high) (C) tumors segmented by quartile *TACSTD2* expression (Q1-Q4). *q < .05. Abbreviations: CRC: colorectal cancer; PDAC: pancreatic ductal adenocarcinoma; UC: urothelial carcinoma.

Next, the association of *TACSTD2* expression levels with the tumor immune landscape was investigated, revealing several significant associations (*P* < .05). A higher prevalence of PD-L1+ tumors was observed in CRC *TACSTD2*-high versus *TACSTD2*-low tumors (6.4% vs 3.9%), with the opposite pattern observed in urothelial cancer (16% vs 37%; Figure 3A). In breast cancer, there was no significant difference in PD-L1 positivity between quartiles in the entire cohort (Figure 3A), and only a small, nonsignificant difference between Q1 (45%) and Q4 (40%) in TNBC (*P *= .06; Supplementary Figure S2).

In CRC, as compared to *TACSTD2*-high tumors, *TACSTD2*-low tumors were enriched in MSI-high (4.8% vs 10.6%) and TMB-high (8% vs 13.6%), respectively (Figure 3B, 3C). These data at first appeared to contradict the known enrichment of MSI-high in CMS1 tumors^20^ and the association of CMS1 with higher *TACSTD2* expression we observed (Figure 1D). However, we speculate that since only 31% of CMS1 tumors in our cohort are MSI-high (Supplementary Figure S3), there is likely a subset of microsatellite stable CMS1 tumors that are *TACSTD2*-high.

**Figure 4.**
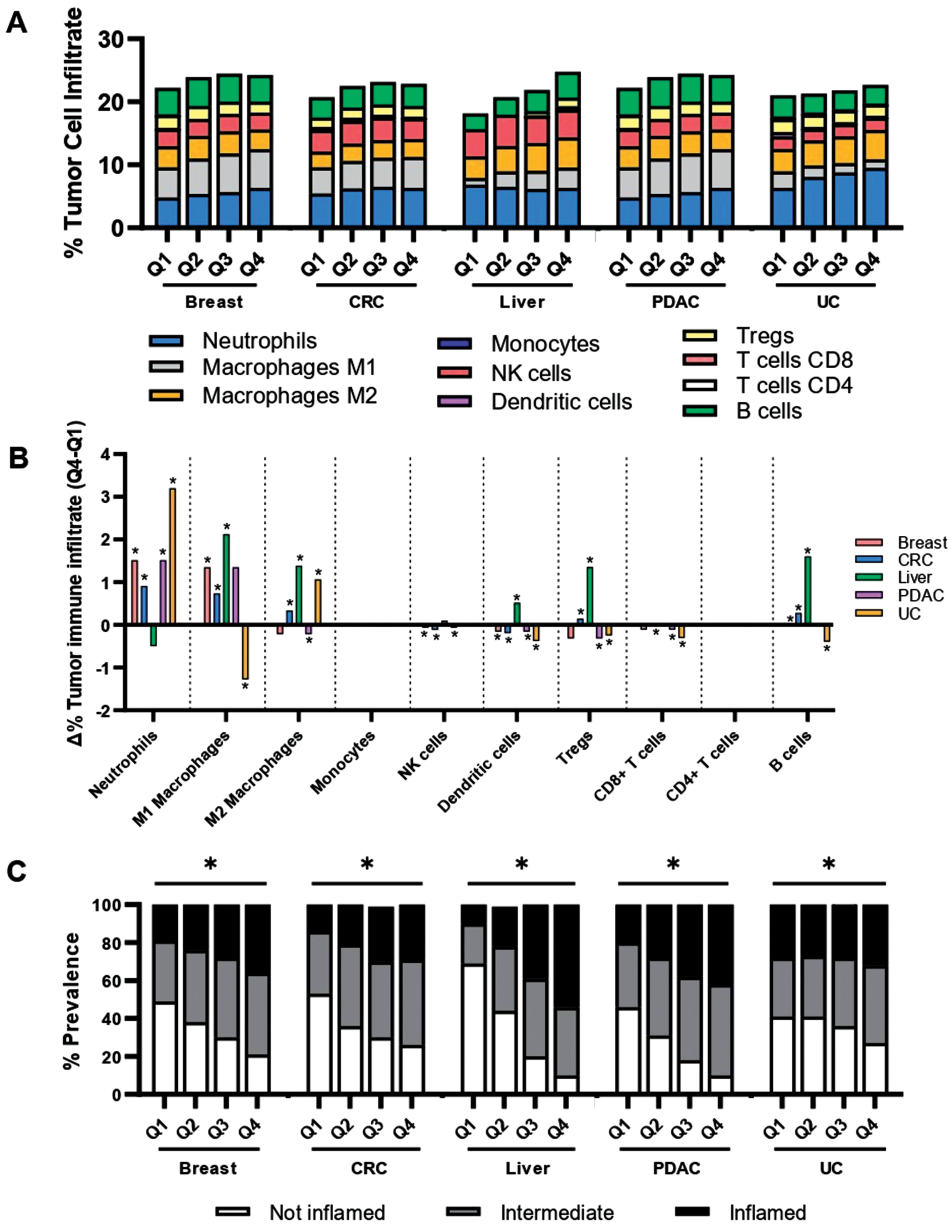
Tumor immune microenvironment of TACSTD2-low versus TACSTD2-high cancer. (A) Immune cell composition based on *TACSTD2* quartile gene expression across tumor types. (B) Difference in immune cell composition between *TACSTD2*-high and *TACSTD2*-low tumors (*q < .05). (C) Prevalence of inflamed tumors (T-cell inflamed score) segmented by *TACSTD2* quartile gene expression (*q < .05). Abbreviations: CRC: colorectal cancer; PDAC: pancreatic ductal adenocarcinoma; UC: urothelial carcinoma.

Next, the immune cell composition of *TACSTD2*-high versus *TACSTD2*-low tumors was inferred using transcriptomic immune cell deconvolution.^16^ A general trend toward greater immune cell infiltration with increasing *TACSTD2* expression levels was observed (Figure 4A). Most notably, neutrophils were significantly elevated in *TACSTD2*-high breast, colorectal, pancreatic, and urothelial cancer, and M1 macrophages were elevated in *TACSTD2*-high breast, colorectal, liver, and pancreatic cancer. *TACSTD2*-high hepatocellular cancer also displayed a greater fraction of M2 macrophages, dendritic cells, T regulatory (T_regs_) cells, and B cells. Of all the cancer types, *TACSTD2*-high urothelial cancer demonstrated the most significant pattern of lower immune cell infiltration, with a smaller fraction of M1 macrophages, dendritic cells, T_regs_, CD8+ T cells, and B cells (*P* < .05; Figure 4B). In addition to immune cell infiltration, *TACSTD2*-high tumors were also more frequently T-cell-inflamed across investigated cancers (breast cancer: 36% vs 19%; CRC: 29% vs 14%; hepatocellular cancer: 54% vs 10%; pancreatic cancer: 42% vs 20%; and urothelial cancer: 32% vs 28%; all *P* < .05; Figure 4C). Together, our data suggests a landscape of greater immune infiltration and inflammation in *TACSTD2*-high tumors compared to *TACSTD2*-low.

### Pan-cancer association of TACSTD2 expression with OS and ICI response

**Figure 5.**
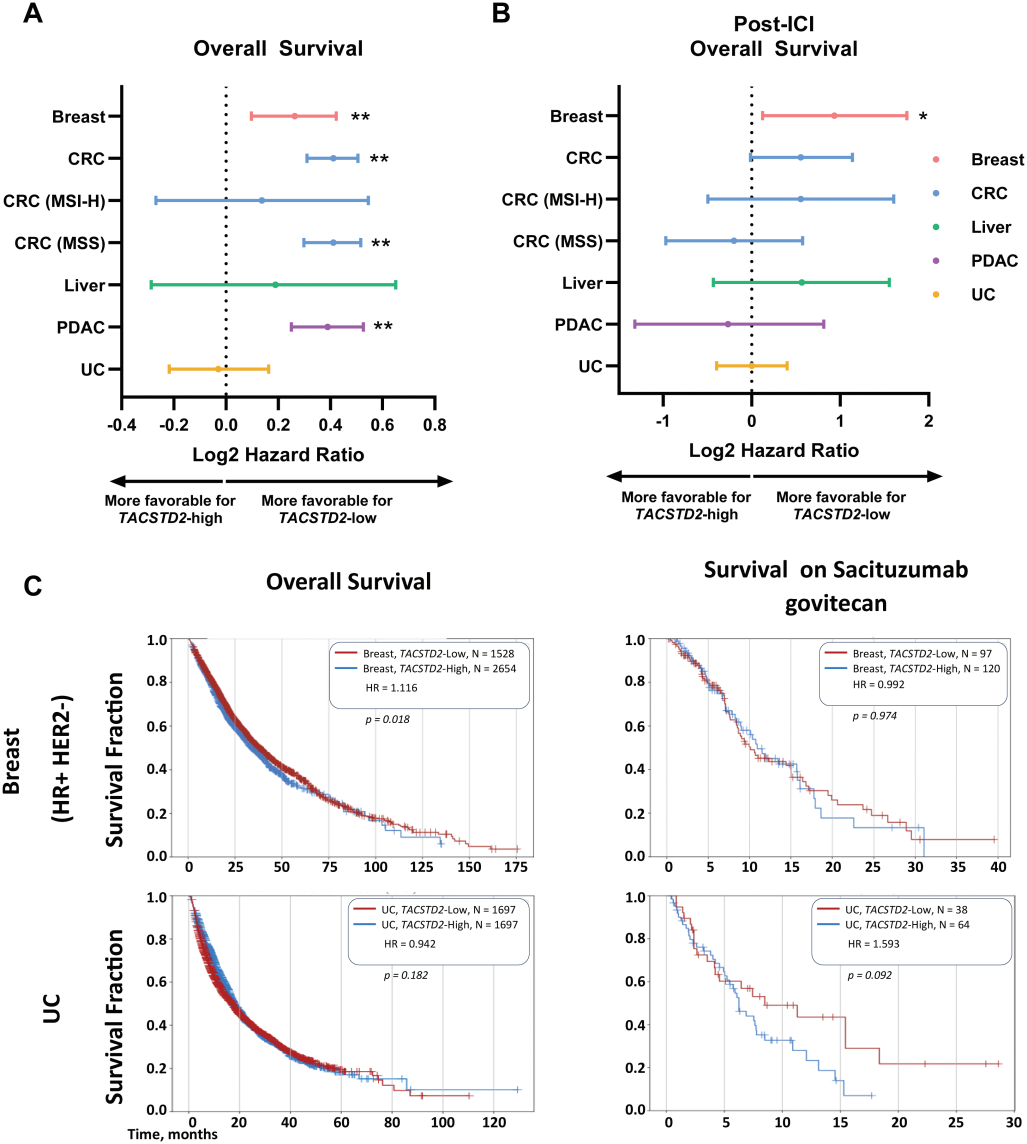
Association of TACSTD2 expression with overall survival (OS) and immune checkpoint inhibitor (ICI) response. Forest plots show log2 hazard ratios (HRs) for OS (A) and post-ICI OS (B) across tumor types. (C) Kaplan-Meier curves for OS and survival on sacituzumab govitecan (SG) for the indicated tumors, higher HRs suggest more favorable OS for TACSTD2-low (*P < .05; **P < .005). OS calculated from tissue collection to last contact; post-ICI OS calculated from first of treatment to last contact and survival on SG calculated from start of treatment to last contact.

The association of *TACSTD2* expression with OS was investigated in each tumor type. *TACSTD2*-high tumors were associated with worse OS in breast cancer (hazard ratio [HR] 1.13 [1.03-1.23], *P* < .007), CRC (HR 1.33 [1.24-1.42], *P* < .001), and pancreatic cancer (HR 1.31 [1.19-1.44]; *P* < .001), while this association was not observed in urothelial or hepatocellular liver cancer (*P* > .05; Figure 5A).

Next, specific subgroups with actionable alterations were investigated. *TACSTD2*-high tumors were associated with worse OS in MSS CRC (HR 1.33 [1.23-1.43], *P* < .001) but not in MSI CRC. In CRC, both *KRAS*-wildtype (wt) and *KRAS*-mutant (mt) groups with *TACSTD2*-high tumors experienced worse OS, while in pancreatic cancer, this was observed only in patients with *KRAS*-mt tumors (Supplementary Figure S4A). When examining the *FGFR3*-mutated urothelial cancer cohort, the *TACSTD2*-high group had better OS than *TACSTD2*-low (HR 0.62 [0.42-0.91]; *P* = .013; Supplementary Figure S4A). These data suggest that even within tumor types, *TACSTD2* may play different roles based on the presence or absence of co-occurring mutations.

Since we observed a differential immune landscape associated with *TACSTD2* expression, OS following treatment with ICI was investigated across tumor types. Only patients with breast cancer with *TACSTD2*-high tumors displayed a shorter post-ICI OS compared to *TACSTD2*-low (HR 1.91 [1.1-3.4]; *P* = .022; Figure 5B). There were no significant differences between *TACSTD2*-low and *TACSTD2*-high in cohorts defined by mutational status (Supplementary Figure S4B).

Finally, OS for HR+ HER2− BC and UC was compared to survival from initiation of SG. TACSTD2-H HR+HER2− BC tumors had significantly worse OS (HR 1.116 [1.019-1.223]; *P* = .018, median survival TACSTD2-H: 34.0; TACSTD2-L: 38.0 months). However, no difference in survival was observed for HR+HER2− BC tumors from the start of treatment with SG to last contact (HR 0.992 [0.679-1.449]; *P* = .974, median survival TACSTD2-H: 10.9; TACSTD2-L: 10.1 months). For UC tumors no difference in OS (HR 0.942 [0.863-1.028]; *P* = .182, median survival TACSTD2-H: 17.9; TACSTD2-L: 17.1 months) nor in OS from start of treatment with SG to last contact (HR 1.593 [0.918-2.765]; *P* = .092, median survival TACSTD2-H: 6.2; TACSTD2-L: 8.4) was observed (Figure 5C).

## Discussion

In this multi-omic analysis of a large pan-cancer cohort, we observed significant tumor type-specific differences in the landscape of molecular alterations as well as a more immune-enriched, or “hot,” microenvironment in *TACSTD2*-high tumors. These findings can illuminate possible approaches to implementing TROP2-directed therapy in combination with other targeted therapies.

ICI in combination with TROP2-directed ADCs may be an attractive option for certain *TACSTD2*-high tumors, such as MSI-high CRC and hepatocellular cancer, where ICI has already seen success. In our CRC cohort, the CMS1 subtype—which is characterized by an MSI-high, immune phenotype^20^—had the highest *TACSTD2* expression, which was in turn associated with more immune infiltration and higher prevalence of T-cell inflamed tumors. Conversely, our cohort showed an association between lower expression of *TACSTD2* and a higher prevalence of MSI-high tumors. At first, these results appeared contradictory, leading us to speculate that there is a subset of microsatellite stable CMS1 tumors that are *TACSTD2*-high.

Further studies on this combination are needed; however, trials like the ongoing trial on the effect of SG combined with pembrolizumab in patients with NSCLC ^21^ and some phase 2 trials have already shown positive results in metastatic urothelial carcinoma^22^ which showed a high response rate in patients who progressed after platinum-based therapy.

While CMS1/MSI-high are biomarkers of ICI response in CRC,^23,24^ liver cancer lacks well-defined biomarkers for ICI response.^25^ Of all the tumor types, liver cancer displayed the most robust increase in immune infiltration and increased prevalence of T-cell-inflamed tumor for *TACSTD2*-high compared to *TACSTD2*-low. Moreover, *TACSTD2*-high liver cancer possessed fewer *CTNNB1* mutations, which are a biomarker for poor ICI response and are associated with an immune “cold” microenvironment.^26,27^ No difference in post-ICI response when segmenting by *TACSTD2*-high versus *TACSTD2*-low was observed even when looking at *CTNNB1* wild-type and mutant tumors separately (data not shown). Although there was no difference in post-ICI response in CRC or hepatocellular cancer, further exploration of *TACSTD2*’s relationship with the tumor immune microenvironment as well as combination approaches of ICI and TROP2 ADC in TROP2+/*TACSTD2*-high tumors may be merited. Another avenue for TROP2 research lies in the development of a new class of trivalent bispecific anti-CD3/anti-TROP2Abs generated using DOCK-AND-LOCK (DNL) technology.^28^ These bispecific antibodies were shown to activate T cells and displayed antitumor activity in a preclinical model of TNBC,^29^ offering another novel option for targeting TROP2 with ICI.

ICI has also seen some success in breast and urothelial cancers. In urothelial cancer, ICI offers an alternative approved treatment for patients who have failed on platinum-based chemotherapy, with ORRs of approximately 20% in patients with PD-L1+ tumors.^30^ In breast cancer, the most prominent results have been observed in PD-L1+ advanced TNBC,^31-33^ leading to the approval of pembrolizumab with chemotherapy for first-line treatment of these patients. Response to ICI in HR+ patients has been rather more disappointing.^34,35^ Collectively, PD-L1+ status has emerged as an important biomarker for ICI response in breast and urothelial cancers; however, we observed no significant differences in PD-L1 positivity associated with *TACSTD2* expression in our cohort. Moreover, while *TACSTD2*-high tumors appeared to exhibit a more immune “hot” microenvironment, patients with breast cancer with *TACSTD2*-high tumors experienced a worse response to ICI. These seemingly contradictory results raise further questions surrounding the biological functions of TROP2 and the landscape of its coalterations, including *TP53* mutations, that may mediate its interaction with the tumor microenvironment.

In this study, there are several actionable alterations that were associated with *TACSTD2* expression and could be considered candidates for targeting by combination therapy. Mutations in *FGFR3* and *ARID1A* were associated with *TACSTD2*-high urothelial cancer, and patients with *FGFR3-*mt *TACSTD2*-high tumors were the only group to experience longer median OS than those with *TACSTD2*-low tumors. These phenomena should be investigated further. *FGFR3* and *ARID1A* mutations are targetable with the pan-FGFR inhibitor erdafitinib^36^ and ATR serine/threonine kinase inhibitors,^37^ respectively. In *TACSTD2*-low breast cancer, FGFR inhibition may also merit further consideration, due to enrichment of *FGF/R* fusions. Moreover, FGFR blockade has been shown to promote immune infiltration in breast cancer,^38^ with this approach under clinical investigation in combination with ICI.^36^ In pancreatic and colorectal cancers, *KRAS* mutations were associated with *TACSTD2*-high tumors, suggesting that these tumors may be more amenable to *KRAS* inhibition with recently developed *KRAS*^G12C^ inhibitors.^39^ The molecularly selected survival analyses also show that the association of *TACSTD2*-high with worse survival in pancreatic and colorectal cancers was not simply related to its association with *KRAS* mutations, since these results were observed in *KRAS-*mt groups as well. Finally, in hepatocellular cancer, *TACSTD2* expression appeared to have the strongest association with the tumor immune microenvironment, but displayed few mutational associations and was not related to prognosis. *CTNNB1* was the only gene mutation significantly associated with *TACSTD2*-low tumors, revealing fewer opportunities for targeted therapy outside of ICI. However, one study found that *CTNNB1*-mutant cells were more responsive to spindle assembly checkpoint kinase (TTK) inhibitors,^40^ suggesting a possible avenue for future research.

The clinical applications of TROP2-targeting have the potential to expand in the near future. Multiple other clinical trials are ongoing, investigating the efficacy of TROP2-targeting ADCs alone or in combination with other targeted therapies in an array of advanced solid tumors.^1,7^ Due to the intrinsic immune-mediated effects of ADCs, ICIs blockade is an attractive treatment companion for these therapies.^7^ However, an unanswered question is if TROP2/*TACSTD2* levels are associated with blockade response. We also briefly explored the outcomes of patients treated with a TROP2 inhibitor (sacituzumab govitecan) retrospectively and found no difference in OS of patients with UC or HR+HER2− BC during the period with the treatment.

The significant strength of this study lies in the cohort size and pan-cancer comparison of *TACSTD2* associations. There are a few limitations, however. While the increase in *TACSTD2* levels in metastatic tissue was small, this difference could nonetheless contribute to the worse survival observed among patients with breast, colorectal, and pancreatic cancer with *TACSTD2*-high tumors. However, our data align with previous studies showing worse prognosis of patients with high TROP2/*TACSTD2* levels.^3^ Furthermore, this cohort lacks stage and grade information, which could possibly affect survival analyses differently in each tumor type. High TROP2 by IHC was found to be associated with advanced stage in CRC, but lower stage in urothelial cancer and lower grade in breast cancer.^41^ Therefore, further studies should incorporate more detailed clinical data in evaluating the association of *TACSTD2* levels with patient outcomes to corroborate our findings. Notwithstanding these limitations, our data strongly points to the association of *TACSTD2* expression with a worse prognosis, a more “hot” tumor immune microenvironment, and a unique mutational landscape among cancers profiled. *FGFR3* and *ARID1A* mutations may represent actionable targets in *TACSTD2*-high urothelial cancer, while *KRAS* mutations may be an attractive target for *TACSTD2*-high CRC and pancreatic cancer. While ICI may have more narrow applications among patients with breast, colorectal, liver, and urothelial cancer, *TACSTD2*-high tumors of these lineages may benefit from ICI in combination with TROP2-directed ADC. Further studies are also needed to elucidate the association of TACSTD2 with ICI resistance-related genes such as KEAP1 and STK11, potentially broadening the strategy of combination therapy of ICI and TROP2-directed ADC.

In summary, the expression of TACSTD2 in diverse solid tumor makes the use of TROP2-directed ADCs an attainable potential target for these tumor types. We expect the target to continue to be clinically actionable and continue to grow in the field, as the landscape of TROP2-directed ADCs broadens. Clinicians ought to be aware of the broad, tumor-agnostic translational relevance of TACSTD2/TROP2 as a potential therapeutic target.

## Supplementary material

Supplementary material is available at *The Oncologist* online.

oyae168_suppl_Supplementary_Material

## Data Availability

The datasets analyzed during the current study are not publicly available but are available from the corresponding author upon reasonable request.
