# Supplementary material for: Genomic, immunologic, and prognostic associations of TROP2 (TACSTD2) expression in solid tumors
Source: Oncologist. 2024 Jul 10;29(11):e1480–91. doi: 10.1093/oncolo/oyae168 (PMC11546728; doi:10.1093/oncolo/oyae168)

Supplementary Materials

Supplementary Figure S1. Pan-tumor expression of TACSTD2 (TPM: transcripts per million). Red line indicates median expression. GIST: gastrointestinal stromal tumor, EOC: epithelial ovarian carcinoma, SCLC: small cell lung cancer, CRC: colorectal cancer, FGTM: female genital tract malignancy, CUP: Carcinoma of unknown primary, NSCLC: non-small cell lung cancer.

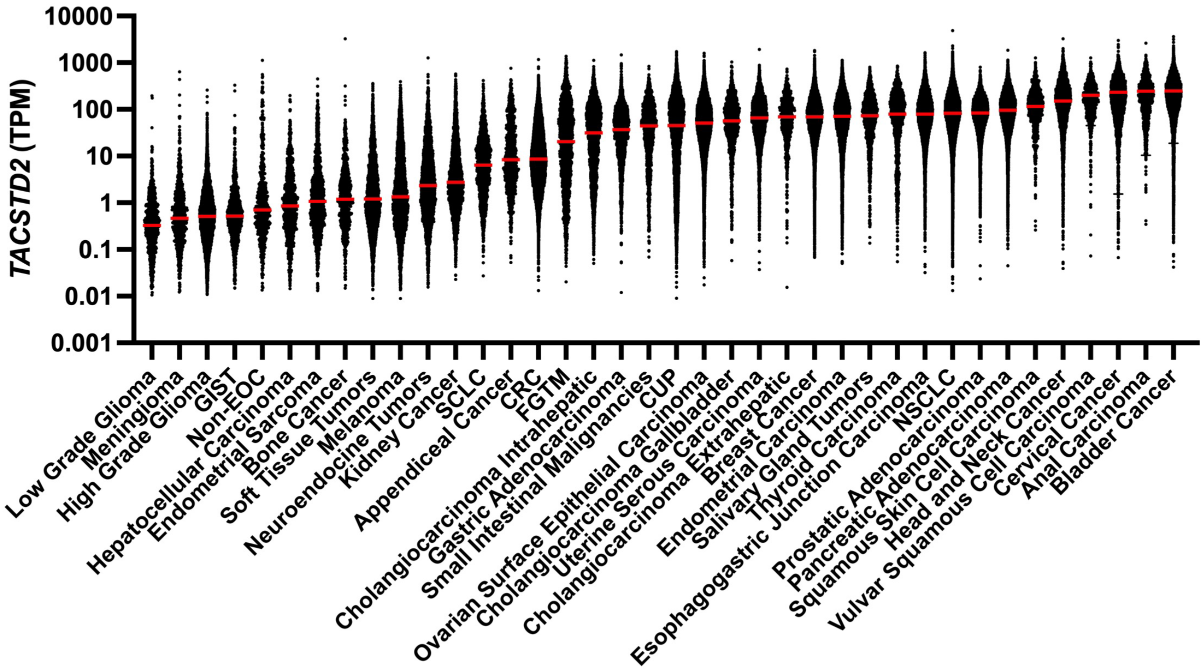

Supplementary Figure S2. PD-L1 positivity in *TACSTD2*-High vs *TACSTD2*-Low TNBC. Percentage of tumors that are PD-L1(+) determined by immunohistochemistry (SP142 antibodyantibody).

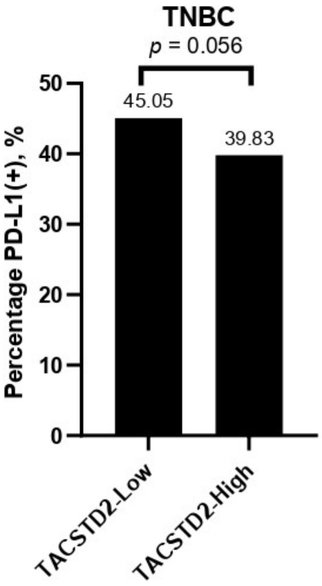

Supplementary Figure S3. Prevalence of MSI-High among each consensus molecular subtype (CMS) in colorectal cancer. Percentage of tumors that are mismatch repair deficient/microsatellite instability-high (dMMR/MSI-High) in each CMS. ****, $q < 0.00005$

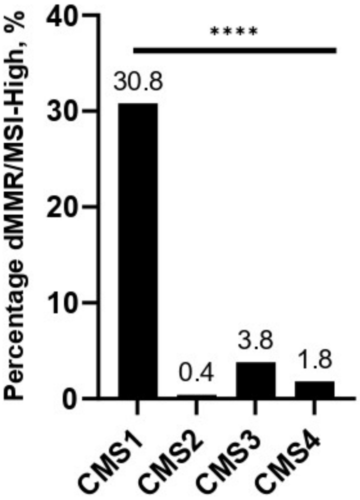

Supplementary Figure S4. Association of *TACSTD2* expression with overall survival (OS) and immune checkpoint inhibitor (ICI) response in molecularly-defined cohorts. Forest plots show log2 hazard ratios for OS **(A)** and post-ICI OS **(B)** across tumor types. Higher hazard ratios suggest more favorable OS for *TACSTD2*-low (**p*<0.05; ***p*<0.005). OS calculated from tissue collection to last contact; post-ICI OS calculated from first of treatment to last contact. CRC=colorectal cancer; PDAC=pancreatic ductal adenocarcinoma; UC=urothelial carcinoma.

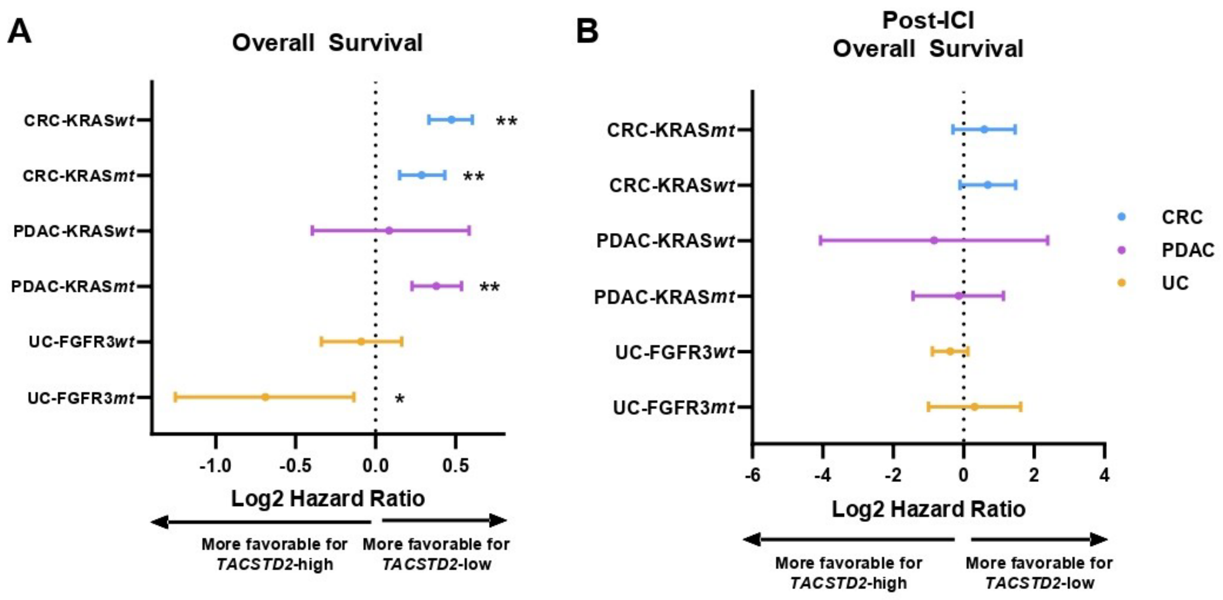

Supplement: oyae168_suppl_Supplementary_Material [file oyae168_suppl_supplementary_material.pdf]
